# Supplementary figures and images for: A pan-cancer comparative analysis of the cancer genome atlas transcriptomic TIL-immune signatures
Source: Cancer Immunol Immunother. 2025 Aug 7;74(9):286. doi: 10.1007/s00262-025-04102-3 (PMC12332167; doi:10.1007/s00262-025-04102-3)

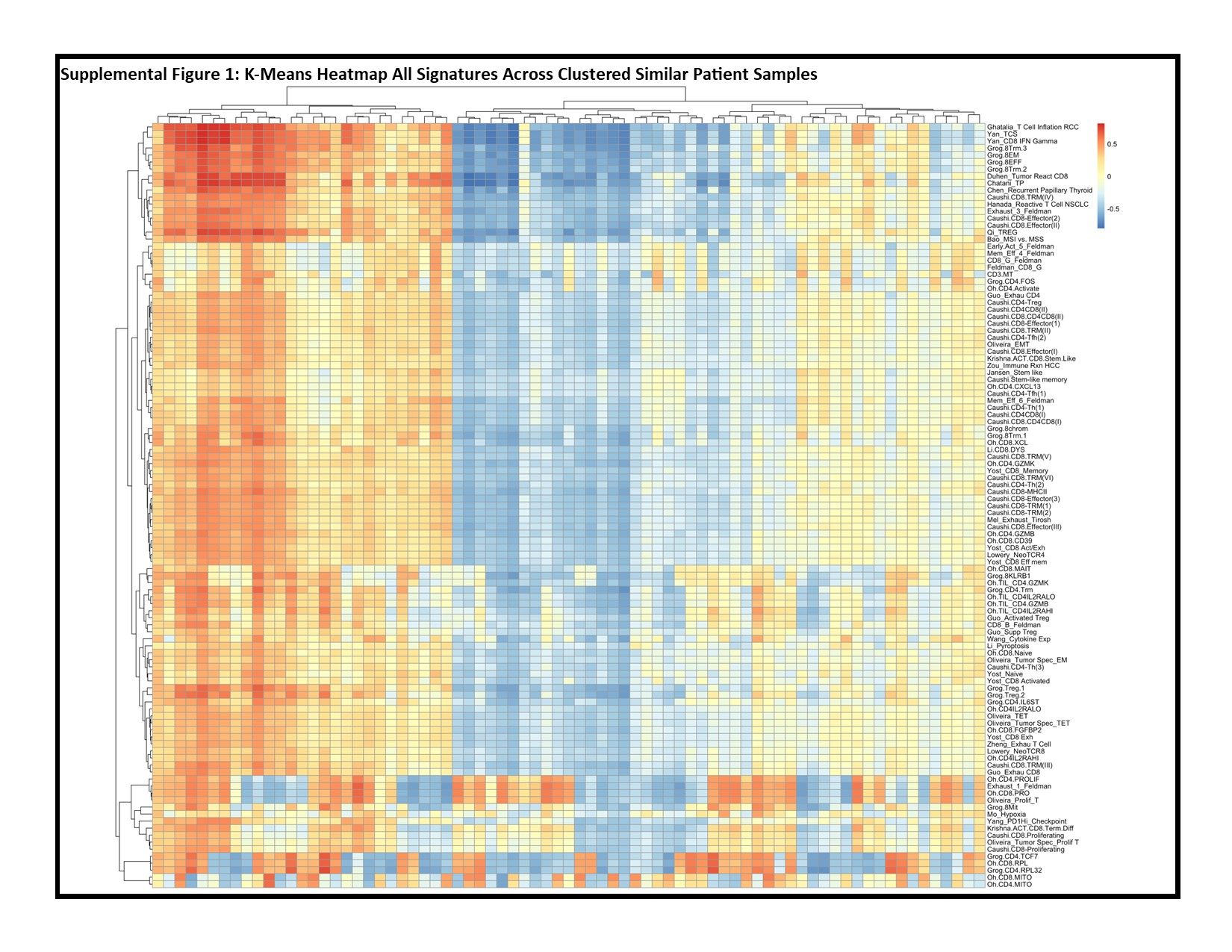

Supplement: Supplementary file 2 — Supplementary file2 (JPG 498 KB) [file 262_2025_4102_MOESM2_ESM.jpg]

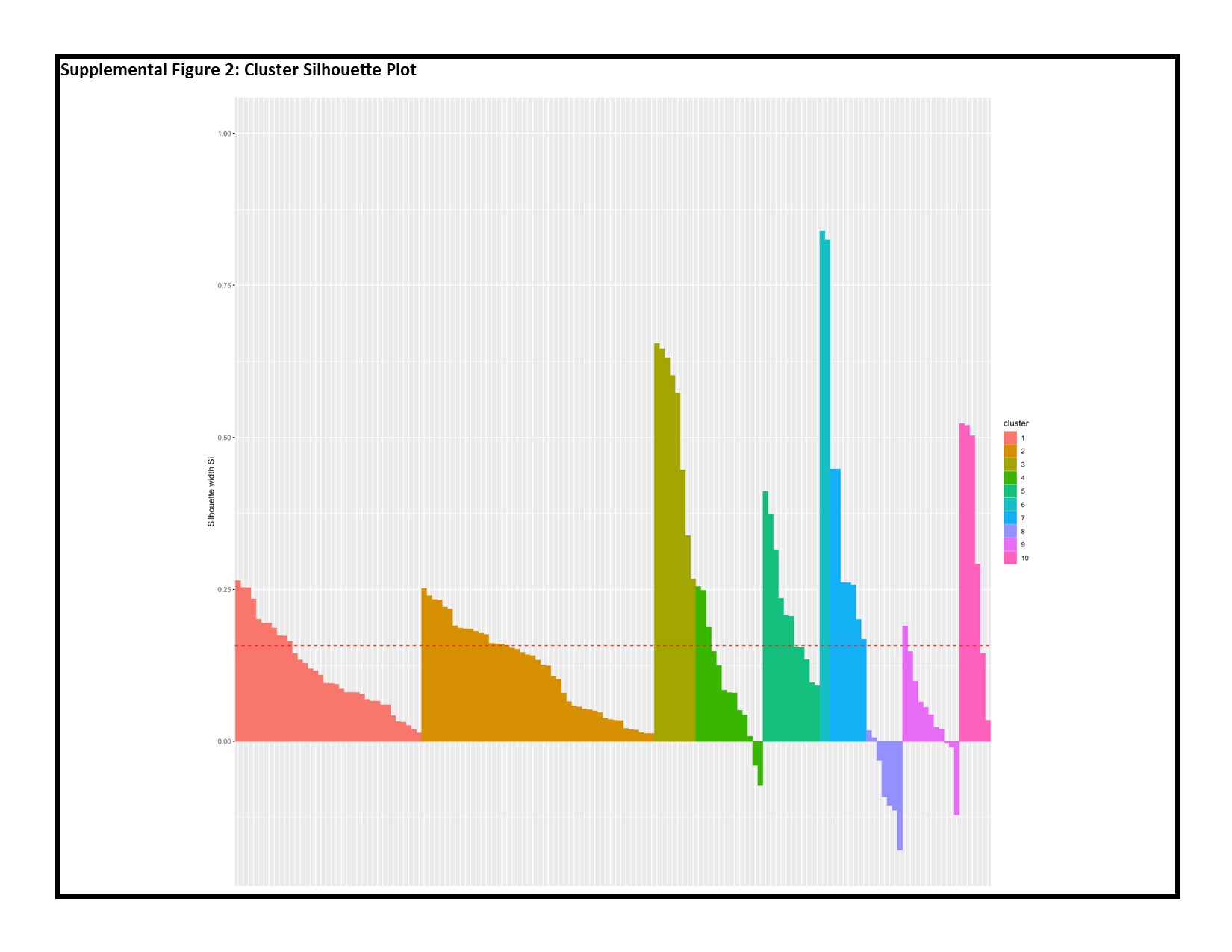

Supplement: Supplementary file 3 — Supplementary file3 (JPG 216 KB) [file 262_2025_4102_MOESM3_ESM.jpg]

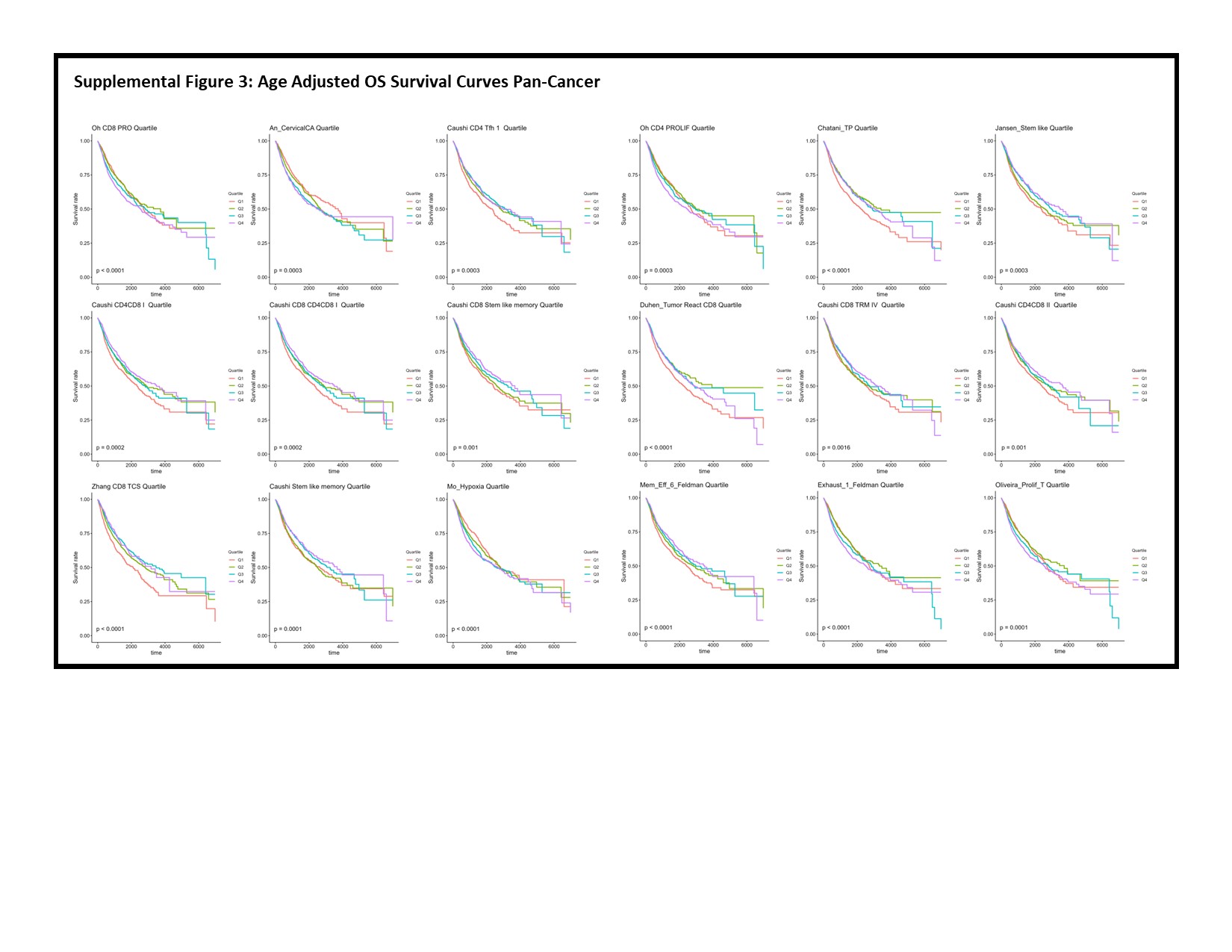

Supplement: Supplementary file 4 — Supplementary file4 (JPG 192 KB) [file 262_2025_4102_MOESM4_ESM.jpg]
